# Surgical Outcomes of Novel Collagen Tile Cesium Brachytherapy for Recurrent Intracranial Tumors at a Tertiary Referral Center

**DOI:** 10.7759/cureus.19777

**Published:** 2021-11-20

**Authors:** Kwanza T Warren, Andrew Boucher, David P Bray, Sean Dresser, Jim Zhong, Hiu-Kuo Shu, Jeffrey Olson, Kimberly Hoang

**Affiliations:** 1 Department of Neurosurgery, Emory University School of Medicine, Atlanta, USA; 2 Department of Neurosurgery, Semmes Murphey Clinic, Memphis, USA; 3 Department of Radiation Oncology, Emory University School of Medicine, Atlanta, USA

**Keywords:** brain metastases, recurrent meningioma, recurrent glioma, gammatile, cesium tile, brachytherapy, recurrent intracranial malignancy

## Abstract

Treatment for recurrent intracranial neoplasms is often difficult and less standardized. Since its approval by the Food & Drug Administration (FDA), GammaTile^TM^ (GT, GT Medical Technologies, Tempe, AZ), a novel collagen tile cesium brachytherapy, has been investigated for use in this population. This study presents the initial experience with the use of GT for patients with recurrent intracranial neoplasms at a tertiary referral center. A retrospective analysis of all patients with GT implantation from November 2019 to July 2021 was performed. Information regarding demographics, clinical history, imaging data, prior tumor treatment, dosing, surgical complications, and outcomes was collected. Twelve patients were included in this study. Pathologies included gliomas (five patients), meningiomas (five patients), and metastatic tumors (two patients). The median tumor volume treated was 24 cc (IQR: 21.2 - 31.3 cc), and patients had a median of 7.5 tiles implanted (IQR: 5.4 - 10.3). One patient had a delayed epidural hematoma requiring reoperation, which was unrelated to GT implantation. Median follow-up was seven months (IQR: 3 -10), with the longest follow-up time of 20 months. Two patients have had local disease recurrence and three patients have had systemic progression of their disease. Three patients are deceased with survivals of 2.9, 4.8, and 5.8 months. Collagen tile brachytherapy is a safe and viable option for patients with recurrent intracranial tumors. Our data are consistent with early results seen at other institutions. Long-term data with larger patient populations are required to assess efficacy, safety, and indications for the use of this novel technology.

## Introduction

While most intracranial neoplasms in adults have well-defined protocols for treatment, depending on tumor characteristics and location, the management of recurrent disease is often less standardized [1-3]. While usually benign, meningiomas can reoccur after primary resection, with WHO grade II/III tumors having significantly higher recurrence rates and shorter progression-free survival (PFS) [2,4]. The management of recurrent meningiomas more often involves radiation therapy as an adjunct to surgical resection when possible, especially depending on the grade of the tumor, with little success seen for systemic agents [5-6]. For glioblastomas, the most common primary malignant brain tumor in adults, treatment at initial diagnosis consists of maximal safe surgical resection followed by adjuvant radiation and temozolomide chemotherapy [7-8]. Virtually all of these tumors reoccur, and treatment can consist of re-resection, repeat radiation, tumor treating fields, and the use of a variety of chemotherapeutic agents [1,9-10]. Treatment of metastatic intracranial lesions is highly dependent on the characteristics of the primary lesion and burden of disease, though patients with recurrent disease often have fewer treatment options and are more likely to receive whole-brain radiation, which is associated with significant morbidity [3,11].

Brachytherapy is a form of radiotherapy that involves the implantation of a sealed radiation source for the delivery of a high dose of radiation with well-defined margins [12]. While brachytherapy is common in the management of several types of cancer, including prostate, breast, and gynecologic neoplasms, it is not currently used as routine treatment for intracranial disease [13-15]. Radiation therapy is the only treatment modality that has been shown to impact survival when used in isolation for patients with malignant brain tumors [16]. While radiation is commonly used in the treatment of newly diagnosed tumors, there has been increasing evidence of the efficacy of re-irradiation at tumor recurrence [17]. Additionally, for patients with glioblastoma, autopsy reports have revealed that there are infiltrative tumor cells up to 2 cm from the visible tumor, indicating that improvement of local control has the potential to impact outcomes of these patients [18-19].

These observations have made brachytherapy an appealing option to use for intracranial neoplasms. Previous studies investigating both intracavitary and interstitial brachytherapy have been taken before for the treatment of malignant neoplasms with a variety of isotopes. Recently, cesium-131 (131Cs) has been established as a promising isotope for this population, given the superior efficacy and safety profile related to a shorter half-life when compared to the previously used permanent iodine-125 (125I) [20-23]. GammaTile^TM^ (GT, GT Medical Technologies, Tempe, AZ) is a form of brachytherapy that utilizes titanium-encapsulated 131Cs seeds embedded in a resorbable collagen-based matrix that gained Food & Drug Administration (FDA) approval for the treatment of newly diagnosed and recurrent brain tumors in 2018. This formulation has several advantages, including ease of implantation, minimization of seed migration after implantation, and reduced risk of radiation necrosis due to lower radiation doses that are released at a slower rate [24].

At our tertiary referral center in Atlanta, Georgia, we have utilized GT for the treatment of recurrent intracranial disease, including gliomas, meningiomas, and brain metastases, since 2019. This paper represents a case series describing clinical characteristics, treatment, and outcomes for patients who have undergone GT implantation.

## Case presentation

Methods

*Patient Population* 

This is a retrospective case series describing patients who underwent GT implantation at our institution between November 2019 and July 2021. Adult patients with recurrent intracranial neoplasms, including primary and metastatic brain tumors, who had exhausted other radiation options were considered appropriate candidates. Patients also had to have a disease that was amenable to good resection with a < 5 mm residual rim and a resection cavity that was an appropriate size for implantation. Patient eligibility was determined in our weekly Adult Brain Tumor Conference in collaboration with Neurosurgery, Neurooncology, Neuroradiology, Neuropathology, and Radiation Oncology providers. The number of GTs to be implanted was estimated preoperatively by radiation oncology based on the anticipated volume and surface area of the resection cavity and radiation dosage required [22].

Implantation Protocol

**Figure 1:**
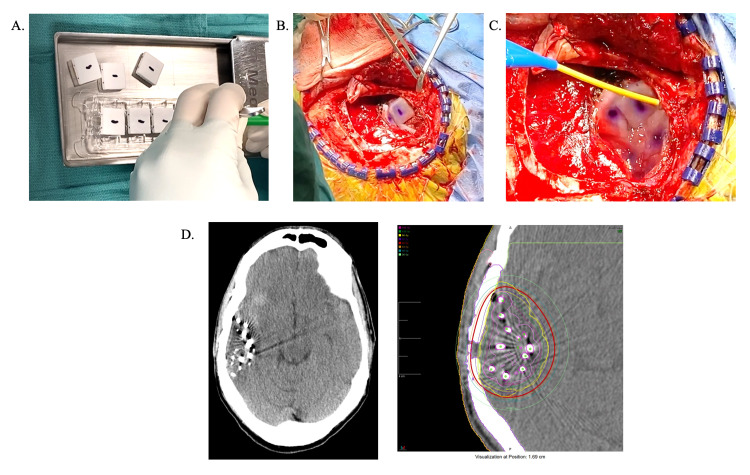
GammaTile Implantation A. GammaTiles pictured here with Cs tiles embedded in the collagen matrix. When implanted, they are inserted with the rough side down. B. After hemostasis is achieved and the resection cavity is irrigated, GammaTiles line the cavity without stacking. C. Fibrin glue can be used to secure the tiles, particularly in larger resection cavities or if there is concern about the mobility of the tiles. D. GammaTiles can be visualized on postoperative CT scan to ensure the cavity is lined and for dosimetry calculations.

Standard craniotomy was performed based on the patient’s tumor type and location. Following maximum safe surgical resection, the presence of tumor was confirmed on frozen section prior to GT implantation by neuropathologists. Hemostasis was achieved and the cavity was irrigated with warmed saline. GTs were used to line the resection cavity without any stacking of tiles. Tiles were cut between the Cs seeds to make them smaller and allow for optimizing their fit into the tumor cavity (Figure 1). The placement of GTs was considered safe in all regions of the brain, including eloquent areas, the posterior fossa, and periventricular areas. We did not implant any tiles adjacent to optic nerves or cranial nerves located in the posterior fossa. In many cases, tissue sealant, such as fibrin glue, was used to keep the tiles in place, particularly in large resection cavities or if there was concern about the mobility of the tiles. Dural closure and skin closure were performed via standard technique based on surgeon preference. All patients had postoperative CT scans within 24 hours of the procedure to confirm coverage and for dosimetry calculations by Radiation Oncology.

Data Collection and Analysis

Demographic factors, including age, sex, race, and tumor type/location, were collected for each patient. As each of these patients had recurrent disease, information regarding tumor treatment prior to GT implantation was collected, including date of diagnosis, prior resections, prior radiation, and prior course of chemotherapy. Data for this intervention included preoperative tumor volume, number of GTs implanted, and postoperative complications. Typically, patients had follow-up visits at three months, six months, nine months, and one-year postoperatively with MRI scans. At each of these time points, information regarding vital status and tumor progression was recorded when available.

All continuous variables are reported using the median, 25th, and 75th percentiles (interquartile range, IQR). Discrete variables are reported with percentages. Given the small sample size, this study was not adequately powered for further statistical testing between variables.

Results

A total of 12 patients had GTs implanted between 2019 and 2021 at our institution. Of these patients, five (41.7%) were male and seven (58.3%) were female. The median age was 62, with an IQR of 46 to 70 (Table 1A). This treatment was used for a variety of intracranial pathologies including gliomas (41.7%), metastatic disease (16.7%), and meningiomas (41.7%) (Table 1B). Eleven (91.7%) of the tumors were supratentorial and the median tumor volume was 24 cc (IQR: 21.2 cc - 31.3 cc) (Table 1C). Ten of these patients had their tumors previously resected, with two of the patients having had three surgical resections prior to GT placement. The two patients who did not have their tumors resected as part of initial management were those with metastatic tumors, both of whom previously received stereotactic radiosurgery (SRS) treatments for their intracranial disease.

**Table 1:** Demographics The table specifies the characteristics of patients included in this study, including demographics (A), primary tumor pathology (B), imaging characteristics (C), and treatments prior to GammaTile placement (D). All continuous variables are reported with medians and 25th and 75th percentiles. Discrete variables are reported as absolute numbers and percentages. WHO = World Health Organization, XRT = radiation therapy

A. Demographics		
Age (med [IQR])		62 (46 - 70)
Sex (n [%])	Male	5 (41.7)
	Female	7 (58.3)
B. Pathology		
Glioma (n [%])	Anaplastic astrocytoma	3 (25)
	Glioblastoma	2 (16.7)
Metastasis (n [%])	Lung adenocarcinoma primary	1 (8.3)
	Breast primary	1 (8.3)
Meningioma (n [%])	WHO Grade II	5 (41.7)
C. Imaging characteristics		
Tumor location (n [%])	Supratentorial	11 (91.7)
	Infratentorial	1 (8.3)
Tumor volume (med [IQR])		24 [21.2 - 31.3]
D. Previous treatments		
Prior resections (n [%])	0	2 (16.7)
	1	6 (50)
	2	2 (16.7)
	3	2 (16.7)
Prior XRT (n [%])	Yes	11 (91.7)
	No	1 (8.3)
Prior chemotherapy (n [%])	Yes	7 (58.3)
	No	5 (41.7)

The median amount of time from the initial diagnosis to GT implantation was 66.8 months (IQR: 35.9 months - 102.8 months). The median number of tiles implanted was 7.5 (IQR: 5.4 - 10.3). Two patients had postoperative complications after the tiles were placed. One patient suffered from symptomatic radiation necrosis. Another patient who underwent craniotomy for resection of a right cerebellar metastatic lesion suffered a delayed epidural hematoma requiring reoperation on postoperative day three (Table 2). This complication was not determined to be related to GT placement. One patient had persistent hydrocephalus postoperatively requiring ventriculoperitoneal shunt placement, though again not thought to be related to GT placement.

**Table 2:** Treatment Characteristics for Patients Who Underwent GT Implantation IQR = interquartile range; CSF = cerebrospinal fluid

Treatment Characteristics		
Months from diagnosis to GammaTile implantation (med [IQR])		66.8 [35.9 -102.8)
Tiles implanted (med [IQR])		7.5 [5.4 - 10.3]
Postoperative complications (n [%])	Symptomatic radiation necrosis	1 (8.3)
	Seizures	0 (0)
	Hemorrhagic complications	1 (8.3)
	CSF leak	0 (0)
	Infection	0 (0)

The median length of follow-up was seven months (IQR: 3 months - 10 months), with the longest follow-up time of 20 months. As of July 2021, five patients (41.7%) have had progression of their disease, with two incidences of local progression (16.6%) and three of systemic progression (24.9%). Median PFS is four months (IQR: 2.5 months - 8.5 months), with medians of 2.5 months for systemic PFS and 10 months for intracranial PFS. In this cohort, three patients are deceased, with survivals of 2.9, 4.8, and 5.8 months, respectively (Table 3). Data regarding the cause of death were not consistently available.

**Table 3:** Clinical Outcomes IQR = interquartile range; PFS = progression-free survival Length of follow-up and PFS are reported in months

Clinical Outcomes		
Length of follow-up (med [IQR])		7 [3 - 10]
Disease progression (n [%])	Local	2 (16.7)
	Systemic	3 (25)
PFS (med [IQR])		4 (2.5 - 8.5)
Vital status (n [%])	Dead	3 (25)
	Alive	9 (75)

## Discussion

In this study, we present our experience with GT implantation and outcomes for patients with recurrent intracranial neoplasms. The large majority of these patients had received prior resections and radiation, causing them to have limited options for treatment of their recurrent disease. In our experience, the implantation process was simple and added minimal time to the operation. We also did not experience any postoperative complications related to GT implantation, and no infections nor wound dehiscence were observed in this population. Two patients had local recurrence of disease while the remainder of patients had progression due to systemic disease progression. It is important to note that the utilization of GT in recurrent tumors is subject to significant bias, as these are patients whose disease had already failed the standard of care and required surgery. This may result in this particular patient population having more aggressive disease and a higher risk of local recurrence.

These findings are consistent with current data regarding GT therapy. A few studies over the past few years have evaluated 131Cs brachytherapy tiles for the treatment of brain metastases [25-28] as well as recurrent meningiomas [29]. The largest of these studies was a prospective trial performed by Wernicke et al. in 2017 and included 42 patients with brain metastases. They demonstrated that 131Cs brachytherapy was able to provide durable local control (89% PFS at one year) with no cases of radiation necrosis, though these patients had not had any previous treatment for their intracranial disease [30]. Results from the same group with a smaller cohort of patients who had previously had radiation treatment of their metastatic intracranial disease demonstrated similar results in terms of efficacy and safety, which is consistent with other similar studies [25,31]. Likewise, Brachman et al. demonstrated the safety and efficacy of 131Cs brachytherapy in a cohort of 19 patients with previously irradiated meningiomas, with only two cases of local progression at 15 months and two cases of radiation necrosis that were amenable to medical management [29]. While none of these studies are sufficiently powered to draw definitive conclusions regarding efficacy when compared to currently used methods for the treatment of recurrent intracranial malignancy, they at least provide promising evidence that it could be a safe and viable option for these patients.

Data for brachytherapy treatment of glioblastoma are less clear. There is concern regarding the effectiveness of brachytherapy for these tumors given the large area of malignant cell infiltration that may extend beyond the radiation margins, making them less likely to prevent local recurrence [24]. Previous studies have evaluated brachytherapy use in newly diagnosed glioblastoma in conjunction with other adjuvant treatments with no real benefit demonstrated [32-33]; one of the studies had to be terminated early due to toxicity. The toxicity could be secondary to the use of 125I radiation; GT 131Cs formulation may be better tolerated from a local toxicity standpoint. Archavlis et al. evaluated brachytherapy in recurrent glioblastoma in conjunction with surgical resection and high-dose temozolomide and did see a survival benefit of three months compared to those who did not receive brachytherapy with comparable complications [34]. Most recently, Wernicke et al. demonstrated that 131Cs brachytherapy has similar efficacy as external beam radiation for recurrent glioblastomas with a lower incidence of radiation necrosis when combined with bevacizumab in a small cohort of 20 patients [35]. While these most recent results are promising, additional properly designed prospective studies and/or meta-analyses must be done to corroborate these findings.

Similar to most of the current studies published that investigate 131Cs brachytherapy for intracranial use, the sample size of this study is too small to draw any definitive conclusions. This is also evident based on the single-institution design and variety of pathologies treated in this small cohort. As an initial cohort, however, this experience has demonstrated the ease of use of GT and the lack of significant morbidity associated with their use. Treating patients in a tertiary care center with a multidisciplinary brain tumor team allows for the safe use of GT brachytherapy. In the future, larger, prospective studies must be performed for patients with recurrent intracranial tumors to better assess their efficacy and safety and move toward the routine use of GT as the standard of care for these patients. Testing this therapy in patients in studies with separation of distinct pathologies and tumor characteristics can help further determine who has the potential to benefit the most from GT therapy.

## Conclusions

Collagen tile brachytherapy is a safe and viable option for patients with recurrent intracranial malignancy. Our data are consistent with early results seen at other institutions. Long-term data with larger patient populations are required to assess the efficacy, safety, and indications for use of this novel technology.
